# Role of the TPR family protein VPA1365 in regulating type III secretion system 2 and virulence in *Vibrio parahaemolyticus*

**DOI:** 10.1128/aem.02201-24

**Published:** 2025-03-25

**Authors:** Wenliang Yin, Mengyan Wan, Youkun Zhang, Hongmei Meng, Zhiming Pan, Xinan Jiao, Dan Gu

**Affiliations:** 1 Jiangsu Key Laboratory of Zoonosis/Jiangsu Co-Innovation Center for Prevention and Control of Important Animal Infectious Diseases and Zoonoses, Yangzhou University 38043 , Yangzhou, Jiangsu, China; 2 Key Laboratory of Prevention and Control of Biological Hazard Factors (Animal Origin) for Agrifood Safety and Quality, Ministry of Agriculture of China, Yangzhou University 38043 , Yangzhou, Jiangsu, China; 3 Joint International Research Laboratory of Agriculture and Agri-product Safety of the Ministry of Education, Yangzhou University 38043 , Yangzhou, Jiangsu, China; The Pennsylvania State University, University Park, Pennsylvania, USA

**Keywords:** *Vibrio parahaemolyticus*, TPR domain, VPA1365, T3SS2, virulence

## Abstract

*Vibrio parahaemolyticus* is a notable seafood-borne pathogen capable of colonizing the intestines of hosts and inducing acute gastroenteritis. The intestinal colonization and enterotoxicity of *V. parahaemolyticus* are highly reliant on the type III secretion system 2 (T3SS2), encoded within the pathogenicity island (Vp-PAI). The expression of Vp-PAI is strictly regulated by bile acid signals and transcriptional regulators VtrA/VtrB. In this study, we identified a tetratricopeptide repeat (TPR) family protein named VPA1365, which regulates the expression of T3SS2 and is indispensable for the intestinal colonization of *V. parahaemolyticus*. The expression and secretion of the T3SS2-dependent protein VopD2 were significantly reduced in Δ*vpa1365* compared to that of the wild type (WT), suggesting that VPA1365 positively regulates the function of T3SS2. Further research indicated that VPA1365 directly binds to the promoters of *vtrA*, thereby increasing the expression levels of T3SS2-associated genes. Additionally, the deletion of *vpa1365* markedly reduced the cytotoxicity, adhesion ability, biofilm formation, and hemolytic activity of *V. parahaemolyticus*. VPA1365 was found to control the expression levels of these virulence-associated genes by binding to the promoters of *scrG*, *pilA*, and *mshA*. In a zebrafish infection model, the Δ*vpa1365* infected groups demonstrated a higher survival rate compared to the zebrafish infected with WT. In conclusion, this study identified a TPR family protein VPA1365, which regulates the expression levels of T3SS2 and virulence-associated genes in *V. parahaemolyticus*, further broadening our understanding of its virulence factors.

**IMPORTANCE:**

The type III secretion system 2 (T3SS2) is of crucial significance for the pathogenicity of *Vibrio parahaemolyticus*; nevertheless, the biological functions of many genes within the T3SS2 gene cluster and the transcriptional regulatory network of T3SS2 remain ambiguous. In this study, we identified VPA1365, a tetratricopeptide repeat family regulator encoded in the T3SS2 gene cluster, which differs from other known T3SS2 regulatory factors, such as OmpR, ToxR, or LysR family proteins. VPA1365 not only positively regulated the expression and secretion of T3SS2-related proteins but also enhanced the virulence in infant rabbits and zebrafish. Moreover, we identified several novel functions of VPA1365, such as its contribution to hemolytic activity, biofilm formation, cytotoxicity, and adhesion ability, uncovering its global physiological role in *V. parahaemolyticus*. The putative VPA1365-binding site was predicted and identified through the MEME-Suite tool and electrophoretic mobility shift analysis. Collectively, these results broaden our understanding of the regulatory pathways of T3SS2 and virulence.

## INTRODUCTION

*Vibrio parahaemolyticus*, widely distributed in the ocean, estuaries, and even freshwater (1), is the causal agent of severe vibriosis in aquatic economic animals (2). *V. parahaemolyticus* infections may lead to diverse life-threatening human diseases, including acute diarrhea and sepsis (3, 4). The pathogenicity of *V. parahaemolyticus* is related to various virulence factors, including the type III secretion system (T3SS1 and T3SS2), thermostable direct hemolysin (TDH), TDH-related hemolysin, type VI secretion system, motility, and biofilm formation (5, 6). During infections, bacteria usually adhere to the surface of the host cell through flagella or pili and then quickly adapt to the host environment by producing polysaccharides to form biofilms, secreting hemolysin, and T3SS effector proteins to evade the host immune system and acquire nutrients (7–9). Interestingly, the fecal samples from 1,900 patients in a Shanghai hospital indicated that the T3SS2, encoded on chromosome 2, was present in all *V. parahaemolyticus* clinical isolates (10). Most strains isolated from the environment only contain the T3SS1 and lack the T3SS2. This finding suggests that T3SS2 may be one of the critical virulence factors in foodborne diseases caused by *V. parahaemolyticus* (11). Thus, it is urgent to understand the pathogenesis mediated by the T3SS2, especially the regulatory mechanisms and biological functions of many hypothetical T3SS2 genes, which is essential for identifying anti-virulence targets and controlling the virulence of the opportunistic pathogen *V. parahaemolyticus* in the future.

The type III secretion system, extensively present in many Gram-negative pathogens, is the needle-like apparatus that injects many effector proteins into host cells to hijack host signaling pathways and quickly cause disease (12–14). The pandemic serotype O3:K6 *V. parahaemolyticus* strain RIMD2210633 generally carries two types of T3SSs (T3SS1 and T3SS2). T3SS1 is regarded as being tightly associated with cytotoxicity, whereas T3SS2 is responsible for bacterial colonization ability and enterotoxicity *in vivo* (15–17). Several functional analyses have shown that the T3SS2 encoded in an 80 kb Vp-PAI plays the major pathogenic factor in tissue invasion and gastroenteritis involved in many animal models (18–20). Various T3SS2 effectors in *V. parahaemolyticus* modifying normal cell functions have been identified, including VopA, VopC, VopG, VopL, VopO, VopT, VopV, VopY, VopZ, and VPA1380 (6, 21–23), which is constantly expanding our knowledge of the enteropathogenicity mechanism of T3SS2 in this bacterium.

The expression of T3SS1 and T3SS2 in *V. parahaemolyticus* is strictly regulated by various environmental signals and transcriptional factors. The AraC/XylR-type transcription factor ExsA regulates the expression of T3SS1 by directly binding to the conserved sequence (5′-TNAAANA-3′) in the promoter region of T3SS1 under the conditions of Dulbecco’s modified Eagle’s medium (DMEM) with low calcium levels (24). Bile salts act as an environmental signal by interacting with VtrA/C, which then VtrA/C activates the expression of the OmpR family transcription factor VtrB. VtrB directly binds to the promoter region of T3SS2 and induces its expression (25, 26). Additionally, the LysR-type transcription factor CalR and transmembrane regulatory protein ToxR could also bind to the promoter region of the gene cluster to directly control T3SS2 transcription (27). Recent studies have shown the essential function of the uncharacterized proteins located in the T3SS2 gene cluster and other environmental factors affecting the function of T3SS2. For example, the gatekeeper protein VgpA (VPA1360) was found to promote the secretion of T3SS2 substrates by interacting with the ATPase VscN2 (28), and a new T3SS2 effector VopY (VPA1324) was proved to suppress host STING-dependent signaling by hydrolyzing c-di-GMP (29). Our previous study discovered that low salinity (0.5% NaCl) induces the secretion of the T3SS2 protein VPA1361, which indicates that low salinity may be a neglected environmental signal regulating virulence of *V. parahaemolyticus* (30). Unfortunately, the regulation patterns of T3SS2 have not been fully elucidated, and the biological functions of several hypothetical proteins in the T3SS2 gene cluster (*vpa1310* to *vpa1396*) require further intensive exploration.

Twenty-two regulators of 230 genes were identified to be involved in the small intestine colonization of *V. parahaemolyticus* by transposon insertion sequencing (Tn-seq) technology (27), including the uncharacterized gene *vpa1365* located on the T3SS2 gene cluster. In this study, we first confirmed that the *vpa1365* indeed contributed to the intestinal colonization of *V. parahaemolyticus*. Moreover, we further investigated the roles of VPA1365 in regulating T3SS2 and the virulence of *V. parahaemolyticus*. Our results indicated VPA1365 positively regulates the expression of numerous genes by binding to the promoters of T3SS2-related and virulence genes, thereby influencing the expression and secretion of T3SS2, hemolytic activity, biofilm formation, adhesion, and cytotoxicity. These findings deepen our understanding of the essential role of VPA1365 in regulating T3SS2 and virulence in *V. parahaemolyticus*.

## MATERIALS AND METHODS

### Bacterial strains, plasmids, and growth conditions

All strains and plasmids utilized in this study were listed and described in Table 1. *Escherichia coli*, *V. parahaemolyticus* RIMD2210633, and its derivative mutants were all grown at 37°C in the Luria-Bertani (LB) medium. The following concentrations of antibiotics were used when necessary: 100 µg/mL carbenicillin (Carb) and 24 µg/mL chloramphenicol (Cm). Furthermore, 1 mM isopropyl β-D-1-thiogalactopyranoside (IPTG) was used to induce the expression and secretion of VPA1365 in the Δ*vpa1365-*vpa1365 and Δ*vpa1365-*pMMB207 strains. The bile salts (0.04%, [wt/vol]) were used to stimulate the expression and secretion of VopD2 in the wild type (WT), ∆*vpa1365*, and complemented strains.

**TABLE 1:** Strains and plasmids used in this study

Strains/plasmids	Description	Reference/source
*E. coli* strains		
DH5α *λpir*	Host for π requiring plasmids	(31)
SM10 *λpir*	Host for π requiring plasmids, conjugal donor	(32)
BL21(DE3)	Host strain for protein expression	Lab collection
BL21(DE3)-*vpa1365*	BL21, pCold1 carrying *vpa1365* open reading frame (ORF), Carb^R^	This study
*V. parahaemolyticus* strains		
RIMD2210633	WT, O3:K6 strain clinical isolate, Carb^R^	(33)
Δ*vpa1365*	RIMD2210633, in-frame deletion in *vpa1365*, Carb^R^	This study
Δ*vscN2*	RIMD2210633, in-frame deletion in *vscN2*, Carb^R^	This study
Δ*vpa1365-*vpa1365	Δ*vpa1365* pMMB207::*vpa1365* (Carb^R^ and Cm^R^)	This study
Δ*vpa1365-*vtrA	Δ*vpa1365* pMMB207::*vtrA* (Carb^R^ and Cm^R^)	This study
Δ*vpa1365-*vtrB	Δ*vpa1365* pMMB207::*vtrB* (Carb^R^ and Cm^R^)	This study
Δ*vpa1365-*pMMB207	Δ*vpa1365* harboring pMMB207 (Carb^R^ and Cm^R^)	This study
Plasmids		
pDM4	Cm^R^, *sacB*, suicide vector that contains an R6K origin of replication (*pir* requiring)	(34)
pMMB207	Cm^R^, IPTG-induced expressing vector	(35)
pCold1	Cm^R^, IPTG-induced expressing vector	Lab collection
pDM4-*vpa1365*	Cm^R^, pDM4 containing homologous arms of *vpa1365* gene	This study
pMMB207-*vpa1365*	Cm^R^, *vpa1365* ORF cloned into pMMB207	This study
pMMB207-*vtrA*	Cm^R^, *vtrA* ORF cloned into pMMB207	This study
pMMB207-*vtrB*	Cm^R^, *vtrB* ORF cloned into pMMB207	This study
pCold1-*vpa1365*	Cm^R^, *vpa1365* ORF cloned into pCold1	This study

### Construction of deletion mutants and complemented strains

The *vpa1365* and *vscN2* (*vpa1338*) in-frame deletion mutants of *V. parahaemolyticus* were generated according to a previous method (36), and the primers were tabulated in Table S1. Briefly, the upstream and downstream fragments of *vpa1365*, amplified from the RIMD2210633 genome DNA with primers *vpa1365* up-F/R and down-F/R, were cloned into the SacI/SalI sites of the suicide vector pDM4 using a *pEASY*-Basic seamless cloning kit (Transgen, Beijing, China). The positive recombinant plasmid pDM4-*vpa1365* was transformed into SM10 *λpir* by heat shock, and then the recombinant plasmid was transferred into the RIMD2210633 strain through conjugation. After that, the double-crossover recombination was selected to produce the in-frame deletion mutant Δ*vpa1365* in the LB agar plate with 10% sucrose. All mutants were confirmed by PCR with the primers *vpa1365* out-F/R and sequenced for reaffirmation. The deletion of the *vscN2* gene in *V. parahaemolyticus* is similar to the above method.

The open reading frame (ORF) of *vpa1365* was amplified with primers *vpa1365*-207-F/R and cloned into the XbaI/HindIII sites of the pMMB207 plasmid (with the chloramphenicol resistance gene). The recombinant plasmids pMMB207::*vpa1365*, pMMB207::*vtrA*, and pMMB207::*vtrB* were transformed into the deletion strain Δ*vpa1365* to generate the complemented strains named Δ*vpa1365-*vpa1365, Δ*vpa1365-*vtrA, and Δ*vpa1365-*vtrB, respectively. The pMMB207 plasmid was transformed into the Δ*vpa1365* to generate the vector control strain named Δ*vpa1365-*pMMB207. All complemented strains were confirmed by PCR with the primers pMMB207-F/R and sequenced.

### Sequence analysis

The National Center for Biotechnology Information accession numbers (http://www.ncbi.nlm.nih.gov/genbank) for VPA1365 and homologous fragments are *V. parahaemolyticus* VPD14 (CP031782.1), *V. parahaemolyticus* VP16 (CP127847.1), *Vibrio cholerae* O17 (AP023371.1), *Vibrio tarriae* 2,521–89 (CP022353.1), *Vibrio mimicus* 07–2,442 (CP046825.1), *Vibrio diabolicus* (CP014037.1), *Vibrio anguillarum* (CP011465.1), *Photobacterium damselae* KC-Na-1 (CP021153.1), and *Grimontia hollisae* 1,078–81 (CP046810.1), respectively. The InterPro tool was used to identify the conserved domains of VPA1365 (https://www.ebi.ac.uk/interpro/), and the 3D protein structure prediction was conducted by SWISS-MODEL (https://swissmodel.expasy.org/). A bootstrap consensus tree was constructed using the neighbor-joining method from the MEGA-X program (37).

### Cytotoxicity and adhesion assay

The lactate dehydrogenase (LDH) released from infected HeLa cells was measured as described previously (38). Overnight cultured *V. parahaemolyticus* strains were adjusted to an OD_600_ of 1, diluted 1:100 into LB medium, and cultured at 37°C with shaking for 4 h. Subsequently, the culture was centrifuged, resuspended, and diluted to a concentration of 10^8^ CFU/mL in DMEM containing 10% fetal bovine serum. HeLa cells were infected by WT, Δ*vpa1365*, Δ*vpa1365-*vpa1365, and Δ*vpa1365*-pMMB207 at a multiplicity of infection of 1:100. After infection at 37°C for 2 h, the LDH cytotoxicity assay was examined and assessed according to the manufacturer’s protocol (Invitrogen, USA). Meanwhile, the infected HeLa cells were lysed with 200 µL of 0.01% Triton-X 100 after washing once with Dulbecco’s phosphate-buffered saline (PBS). The 10-fold serial dilution lysates (100 µL) were respectively plated on thiosulfate citrate bile salt sucrose agar plates culturing at 37°C for 18 h. The adhesion rate was calculated as the number of bacterial cells adhered per the input total number of bacterial cells.

### Hemolytic activity assay

Overnight cultures of WT, Δ*vpa1365*, Δ*vpa1365-*vpa1365, and Δ*vpa1365*-pMMB207 were centrifuged, washed twice with PBS, resuspended in PBS at a final concentration of 1 × 10^9^ CFU/mL, and then spotted 10 µL on the Wagatsuma blood agar base for 37 h. The measured circle diameter of the clear zone was considered as the hemolytic activity (39, 40). Three separate experiments were performed in biological triplicates each.

### Biofilm formation assay

Overnight cultures of WT, Δ*vpa1365*, Δ*vpa1365-*vpa1365, and Δ*vpa1365*-pMMB207 were adjusted to an OD_600_ of 1, diluted 1:100 into 200 µL of fresh LB medium in the 96-well polystyrene microplate, and cultured statically at 37°C for 31 h. The bacterial cells were removed and washed twice with PBS, and 220 µL of 0.1% crystal violet was added to stain biofilm for 30 min. Afterward, 200 µL of 95% ethanol was added to dissolve the crystal violet for 1 h after removing residual stains, and the amount of biofilm was determined at 570 nm using a microplate reader (Epoch 2, BioTeK). The biofilm formation was normalized as the total biofilm per cell density of bacteria. Three separate experiments were performed with biological triplicates each.

### Immunoblotting analysis

For VopD2 immunoblotting analysis was carried out as previously reported (30), the overnight cultures of WT, Δ*vpa1365*, Δ*vpa1365-*vpa1365, Δ*vpa1365*-pMMB207, Δ*vpa1365*-vtrA, and Δ*vpa1365*-vtrB were diluted 1:100 into 5 mL of fresh LB medium and cultured under different environmental factors (at 28°C, 40°C, pH = 8, 0.1 M NaCl, 0.5 M NaCl, 37°C with or without 0.04% bile salts) for 7 h, respectively. Next, the bacterial cultures were adjusted to an OD_600_ of 1, the supernatant protein was collected and concentrated using protein ﬁlter columns Amicon Ultracel and Regenerated Cellulose (Millipore, Billerica, MA, USA), and cell pellets were resuspended in 100 µL PBS. The 5× SDS-PAGE sample loading buffer (Beyotime, Shanghai, China) was added to the mixture, and then each 10 µL normalized sample incubated at 100°C for 10 min was separated on a 10% polyacrylamide gel and transferred to the nitrocellulose filter membranes (Solarbio, Beijing, China). After blocking in 1% bovine serum albumin for 1.5 h, the nitrocellulose filter membranes were incubated with the VopD2 antibody or RNA polymerase (RNAP) antibody at a 1:1,000 dilution and then incubated with goat anti-rabbit IgG (Sangon, Shanghai, China) at a 1:4,000 dilution. Finally, the SuperFemto ECL chemiluminescence reagent (Vazyme, Beijing, China) was used to visualize each blot.

### EMSA assay

The ORF region of *vpa1365* was amplified and inserted into the pColD1 plasmid with the EcoRI and BglII sites, and then the recombinant plasmid pColD1-*vpa1365* was transformed into *E. coli* BL21 (DE3). The primers are listed in Table S1. IPTG was added to induce the expression and secretion of VPA1365 growing in 500 mL of LB medium at 16°C with 120 rpm for 16 h; subsequently, the cultures were collected, washed, lysed, and subjected to the purification of VPA1365-His protein using the His Bind Purification Kit (Sangon, Shanghai, China). All purified proteins were examined for concentration using a bicinchoninic acid assay kit (Beyotime, Shanghai, China). The DNA probes with the 5′-FAM fluorescent label were amplified and purified using the Gel Extraction Kit (Omega, USA). Each 20 µL of a different electrophoretic mobility shift (EMSA) mixture containing 30 nM of DNA-purified probes, 4 µL of 5× EMSA binding buffer (10 mM NaCl, 0.1 mM dithiothreitol, 0.1 mM EDTA, 10 mM Tris, pH 7.4), and the VPA1365-His protein was individually incubated at 25°C for 0.5 h and separated on a 6% precast PAGE gel (Beyotime, Shanghai, China). When necessary, unlabeled cold DNA was added to the mixture. Finally, Typhoon FLA 9500 was used to visualize the imprint (GE Healthcare, Uppsala, Sweden).

### Quantitative real-time reverse transcription PCR analysis

Quantitative real-time reverse transcription PCR (qRT-PCR) was performed as described previously (41). In brief, the overnight cultures of WT, Δ*vpa1365*, Δ*vpa1365-*vpa1365, and Δ*vpa1365-*pMMB207 were grown in LB medium with 0.04% bile salts to the stationary phase, the total RNA was extracted with the bacterial RNA extraction kit (Tiangen, Beijing, China), and the RNA quality was measured by Nanodrop (Nanophotometer NP80, IMPLEN, München, Germany). All normalized samples were treated with the HiScript III All-in-One RT SuperMix kit (Vazyme, Nanjing, China) at 50°C for 15 min and then at 85°C for 15 s to reverse transcription into cDNA. The cDNA samples were determined and analyzed on the ABI PRISM 7500 Real-Time PCR System (Applied Biosystems, USA) with the ChamQ Universal SYBR qPCR Master Mix (Vazyme, Nanjing, China). Expression levels of the genes encoding T3SS2 apparatus protein VPA1342 and VPA1343, regulator protein VPA1348 (VtrB), translocator protein VPA1361 (VopD2), hypothetical protein VPA1364, effector VPA1380, type IV pilin MshA and PilA, thermostable direct hemolysin TdhA and TdhS, biofilm regulatory protein ScrG, and capsular polysaccharide-protein CpsA were determined. Primers in this study are listed in Table S1. The relative mRNA expression values of each gene normalized to *gyrB* gene expression were calculated by using the 2^−ΔΔCt^ method (42). Three separate experiments were performed in biological triplicates each.

### Animal infection

First, the infant rabbit infection model was performed to determine the bacterial T3SS2 function and intestinal colonization assay (43). Overnight cultures of WT and Δ*vpa1365* were diluted 1:100 into 50 mL of fresh LB medium and cultured at 30°C for 16 h. Infant rabbits were infected with WT and Δ*vpa1365* at a dose of 10^9^ CFU orally, and colonized bacteria were counted by enumerating the CFU in 1 g of the small intestine tissues with gradient dilution. Three infected tissues in each group were submitted to Wuhan Servicebio Technology Co. Ltd. for pathological section analysis. The representative image was respectively presented. Besides, the zebrafish infection model was used to evaluate the effect of VPA1365 on bacterial virulence (44). Healthy *Danio rerio* (zebrafish, 2–2.5 cm in length) was raised in freshwater at the constant 26°C ± 2°C for 1 week before infection. Each group of 30 zebrafish cultured at constant temperature was infected with intramuscular injection at a dose of 10^6^ CFU per 5 µL of the WT, Δ*vpa1365*, and Δ*vscN2*, respectively, afterward observed and recorded the number of living zebrafish every day.

### Statistical analysis

All statistics were analyzed with GraphPad Prism version 7.01, and the results were summarized as mean ± SD (*n* = 3). The one-way analysis of variance with Dunnett’s post-test or *t*-test was used to determine the differences by calculating *P* value. The *P* values ≤0.05 and ≤0.01 were considered significant (*) and extremely significant (**), respectively. The *P* value > 0.05 was regarded as non-significant.

## RESULTS

### Identification and sequence analysis of vpa1365

**Fig 1:**
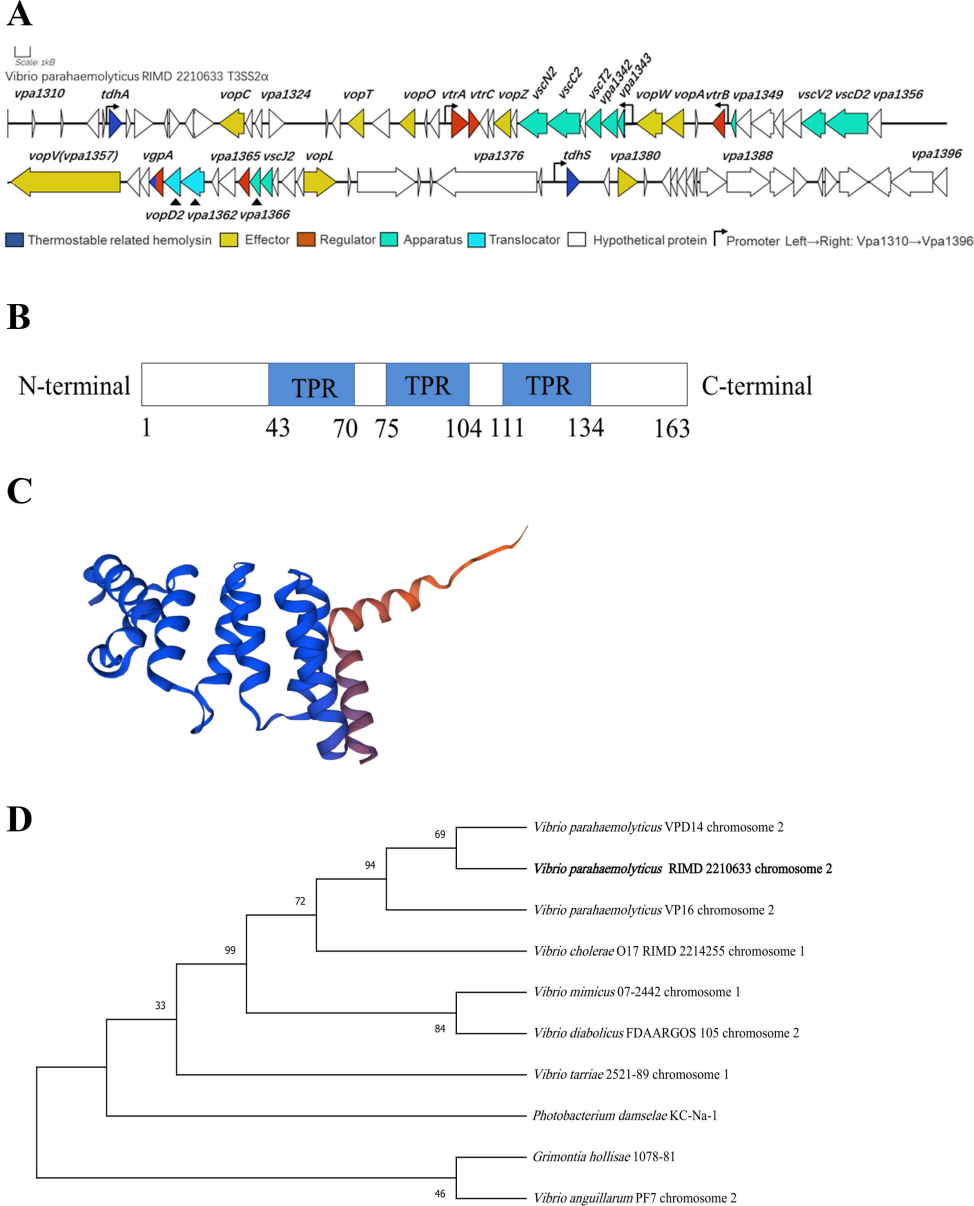
Conserved domain structure and phylogenetic tree analysis of VPA1365 in *V. parahaemolyticus*. (**A**) Genetic location and organization of the T3SS2 in *V. parahaemolyticus* RIMD 2210633. (**B**) Three blue boxes represent the conserved TPR domain predicted by the INTERPRO. (**C**) The VPA1365 structure prediction was accomplished using the SWISS-MODEL (the blue and orange regions indicate the amino terminal and carboxy terminal, respectively). (**D**) The phylogenetic tree inferred from 1,000 replicates was constructed by the neighbor-joining method using MEGA-X.

Nucleotide sequence analysis showed that the ORF of *vpa1365* contains 489 base pairs, encoding 163 amino acids within the T3SS2 gene cluster located in the small chromosome of *V. parahaemolyticus* RIMD2210633 (Fig. 1A). The INTERPRO and UnitProtKB analyses indicated that the VPA1365 protein contains three conserved tetratricopeptide repeat (TPR) domains, which mediate protein-protein and protein-DNA interactions (45–48), suggesting a potential role as a regulator (Fig. 1B). The structural prediction of the VPA1365 protein using SWISS-MODEL revealed a 90.68% similarity to the structure of a regulatory factor called A0A7Z7VL62_VIBCL found in *V. cholerae* (Fig. 1C). Additionally, phylogenetic tree analysis indicated that the sequence of *vpa1365* in *V. parahaemolyticus* shares a relatively high degree of similarity with other *Vibrio* species (60%–85% amino acid identity; Fig. 1D). These findings collectively suggested that the uncharacterized VPA1365 might be a highly conserved TPR family protein commonly present in *Vibrio* species.

### Deletion of vpa1365 reduces the intestinal colonization of *V. parahaemolyticus*

**Fig 2:**
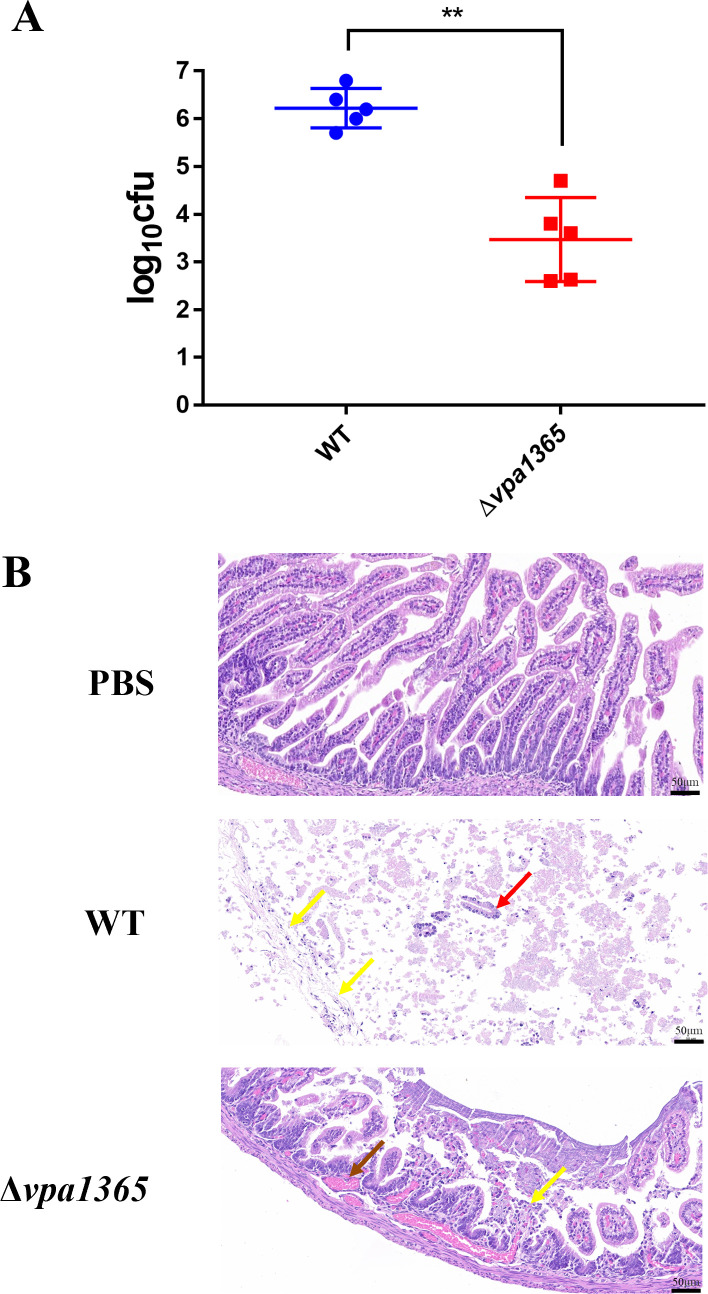
Analysis of the intestinal pathological section and bacterial colonization quantity in infant rabbits. (**A**) The amount of WT and Δ*vpa1365* colonized in the small intestine. **P ≤ 0.01. (**B**) Observations on the small intestine were challenged with PBS, WT, and Δ*vpa1365*, respectively. The yellow arrow, red arrow, and brown arrow represented exfoliated intestinal villous epithelial cells, damaged lamina propria, and vascular congestion, respectively. Three infected tissues from each group were analyzed, and a representative image was presented for each case. Scale bars: 50 µm.

To further elucidate the function of VPA1365 in infant rabbit intestinal colonization, we generated the in-frame gene deletion mutant strain Δ*vpa1365*, as well as complement strains Δ*vpa1365-*vpa1365, Δ*vpa1365*-pMMB207, Δ*vpa1365-*vtrA, and Δ*vpa1365-*vtrB. The growth curve results showed that no significant differences were observed among the WT, Δ*vpa1365*, and complement strains when cultured at 37°C in the LB liquid medium (Fig. S1). However, after infection for 38 h, the bacterial burden in the Δ*vpa1365*-infected small intestine of infant rabbits was significantly lower than that of the WT-infected infant rabbits (Fig. 2A; *P* ≤ 0.01). Moreover, the pathological analysis indicated that the unchallenged intestinal tissue exhibited long intestinal villi and an intact villous epithelium composed of columnar epithelial cells. Intestinal tissue infected with Δ*vpa1365* displayed short villi, increased shedding of villous epithelial cells, and an exposed lamina propria, indicating a slight injury to the tissue (Fig. 2B). However, widespread and obvious shedding of intestinal villi was observed in the intestinal tissue challenged with WT. The intestinal glands in the lamina propria, the structures of the lamina propria, and the muscle layers were not obvious, suggesting that the intestinal tissue suffered severe injury challenged with WT (Fig. 2B). These findings showed that *vpa1365* did play an important part in the enterotoxicity and intestinal colonization of *V. parahaemolyticus*.

### VPA1365 regulates the expression and secretion of T3SS2 by directly binding to the promoters of vtrA

**Fig 3:**
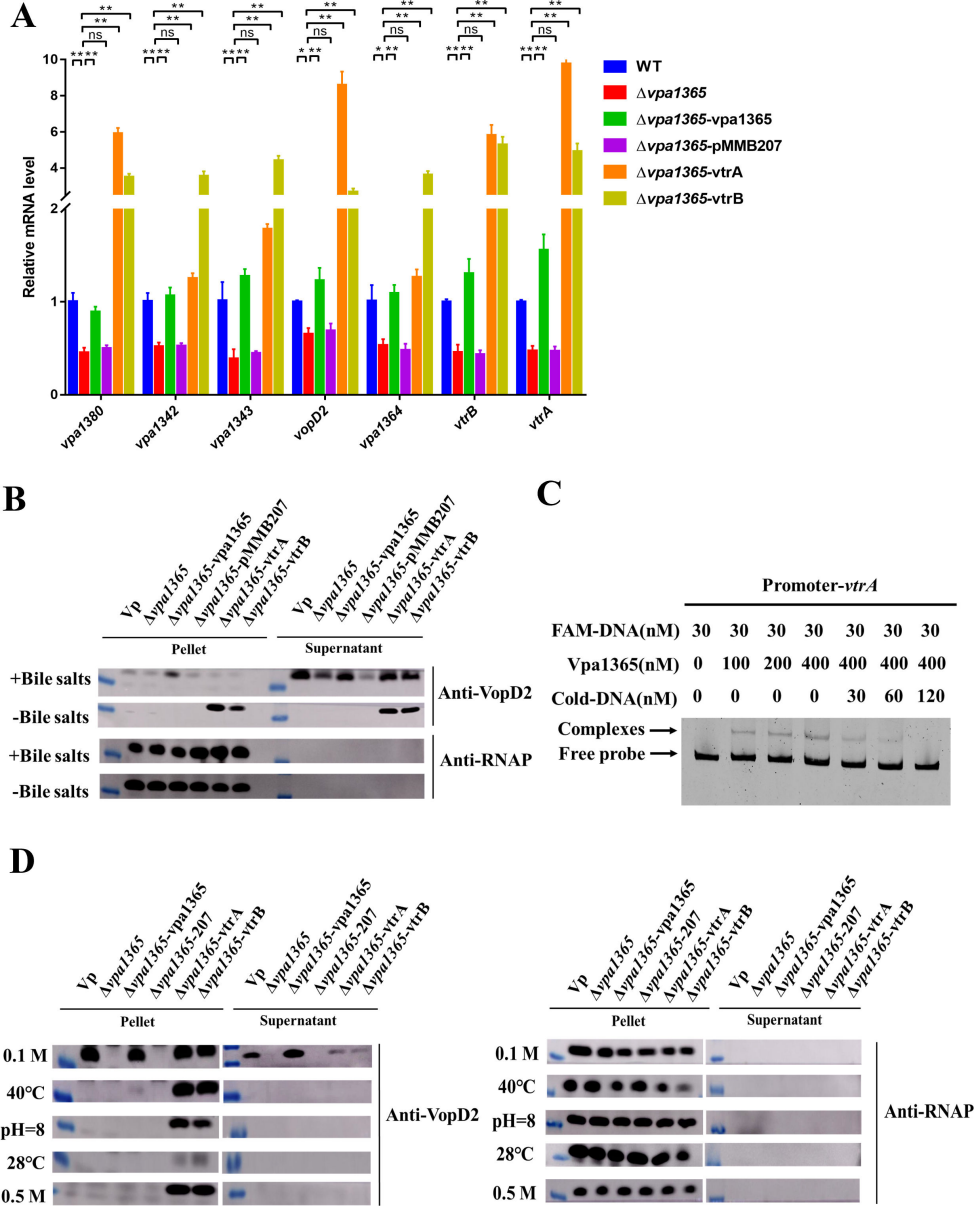
The effects of *vpa1365* on the expression and secretion of T3SS2. (**A**) Relative transcript levels of the T3SS2-associated genes in WT, ∆*vpa1365*, ∆*vpa1365-*vpa1365, ∆*vpa1365-*vtrA, and ∆*vpa1365-*vtrB. Results are represented as mean ± SD (*n* = 3, biologically independent experiments). *P ≤ 0.05 and **P ≤ 0.01. ns, no significance. (**B**) The T3SS2 translocon protein VopD2 secretion and RNAP of WT, ∆*vpa1365*, and complemented strains in LB medium with or without 0.04% bile salts. (**C**) The analysis of binding of VPA1365 to the promoter of the regulator *vtrA*. (**D**) The T3SS2 translocon protein VopD2 secretion and RNAP of WT, ∆*vpa1365*, and complemented strains in LB medium at 28°C, 40°C, pH = 8, 0.1 M NaCl, and 0.5 M NaCl, respectively.

It is generally considered that *V. parahaemolyticus* T3SS2 is induced by bile salts and responsible for enterotoxicity and intestinal colonization (11, 26). Therefore, we evaluated the expression levels of several important T3SS2 genes in WT, Δ*vpa1365*, Δ*vpa1365-*vpa1365, and Δ*vpa1365-*pMMB207 in the presence of bile salts. Transcription levels of multiple T3SS2-related genes (*vpa1342*, *vpa1343*, *vtrB*, *vopD2*, *vpa1364*, and *vpa1380*) were significantly decreased in Δ*vpa1365* and Δ*vpa1365-*pMMB207 compared to that of WT, and the expression levels of these genes were restored in the Δ*vpa1365-*vpa1365 (Fig. 3A; *P* ≤ 0.05). VopD2 has been described as a translocator protein secreted by the T3SS2, which serves as an indicator of T3SS2 functionality (49). Furthermore, we subsequently assessed the secretion of T3SS2 protein VopD2 in WT, Δ*vpa1365*, Δ*vpa1365-*vpa1365, and Δ*vpa1365*-pMMB207 cultured in LB medium with 0.04% bile salts. An RNAP antibody was used to demonstrate that the samples were evenly loaded by assessing RNAP levels. As expected, the secretion of VopD2 in the WT was signiﬁcantly higher than that in both Δ*vpa1365* and Δ*vpa1365*-pMMB207 (Fig. 3B), and the complementary strain Δ*vpa1365*-vpa1365 recovered the VopD2 secretion to WT levels. These findings indicated that VPA1365 positively regulates the expression and secretion of the T3SS2 proteins in *V. parahaemolyticus*.

To further investigate the potential regulatory role of VPA1365 on the T3SS2, EMSA was performed to observe the binding of VPA1365 to the promoter of the T3SS2 effector gene *vpa1380*, hypothetical protein gene *vpa1364*, and regulator genes *vtrA* and *vtrB in vitro*. The results showed that the VPA1365 protein binds to the promoters of *vtrA* in a concentration-dependent manner, and the addition of unlabeled cold DNA resulted in a reduced shifted band (Fig. 3C). While no binding is observed to the promoter of the *vtrB*, *vpa1380*, and *vpa1364* (Fig. S2B through D). The *gyrB* probe was used as a negative control, showing no binding with the VPA1365 protein (Fig. S2A). These results suggested that the VPA1365 protein binds directly to the promoter regions of *vtrA* to activate the expression of T3SS2-related genes, thereby positively affecting the virulence of *V. parahaemolyticus*.

Environmental factors have been found as a component to induce the expression and secretion of the T3SS2. To further explore this, we analyzed the expression and secretion of VopD2 under various environmental conditions (28°C, 40°C, pH = 8, 0.1 M NaCl, 0.5 M NaCl). As expected, the expression and secretion of VopD2 were observed in WT under the condition of 0.1 M NaCl (Fig. 3D), which is consistent with our previous findings (30). Meanwhile, the deletion of *vpa1365* abolished the expression and secretion of VopD2 under low NaCl conditions. The complementary strain Δ*vpa1365*-vpa1365 was restored to the level of WT (Fig. 3D), further confirming the regulation of VPA1365 to T3SS2.

Our study demonstrated that VPA1365 inhibits the expression of *vtrA*, a key activator of T3SS2 in *V. parahaemolyticus* (50). This led us to investigate whether VPA1365 regulates T3SS2 expression via VtrA. To explore this, we overexpressed VtrA or VtrB in Δ*vpa136* and assessed the expression levels of T3SS2-related genes using qRT-PCR, while VopD2 secretion was evaluated by western blotting. As expected, overexpression of either VtrA or VtrB in Δ*vpa1365* significantly increased the expression levels of T3SS2-related genes (Fig. 3A; *P* ≤ 0.01), which indicates that overexpression of VtrA or VtrB suppresses the reduced expression of T3SS2 and secretion of VopD2 in Δ*vpa1365*. Additionally, overexpressing VtrA or VtrB in Δ*vpa1365* restored the expression of VopD2 in the whole cell under all tested environmental conditions. However, the secretion of VopD2 in Δ*vpa1365*-vtrA or Δ*vpa1365*-vtrB strains was observed under low NaCl conditions, and the secretion levels were lower than those in the WT and complementary strains (Fig. 3D). These results revealed the function of VPA1365 in sensing the environmental cue (low NaCl) and extensive regulatory connections between the T3SS2 positive regulator VPA1365 and the VtrA/VtrB.

### Deletion of vpa1365 reduces the cytotoxicity and adhesion rate to HeLa cells

**Fig 4:**
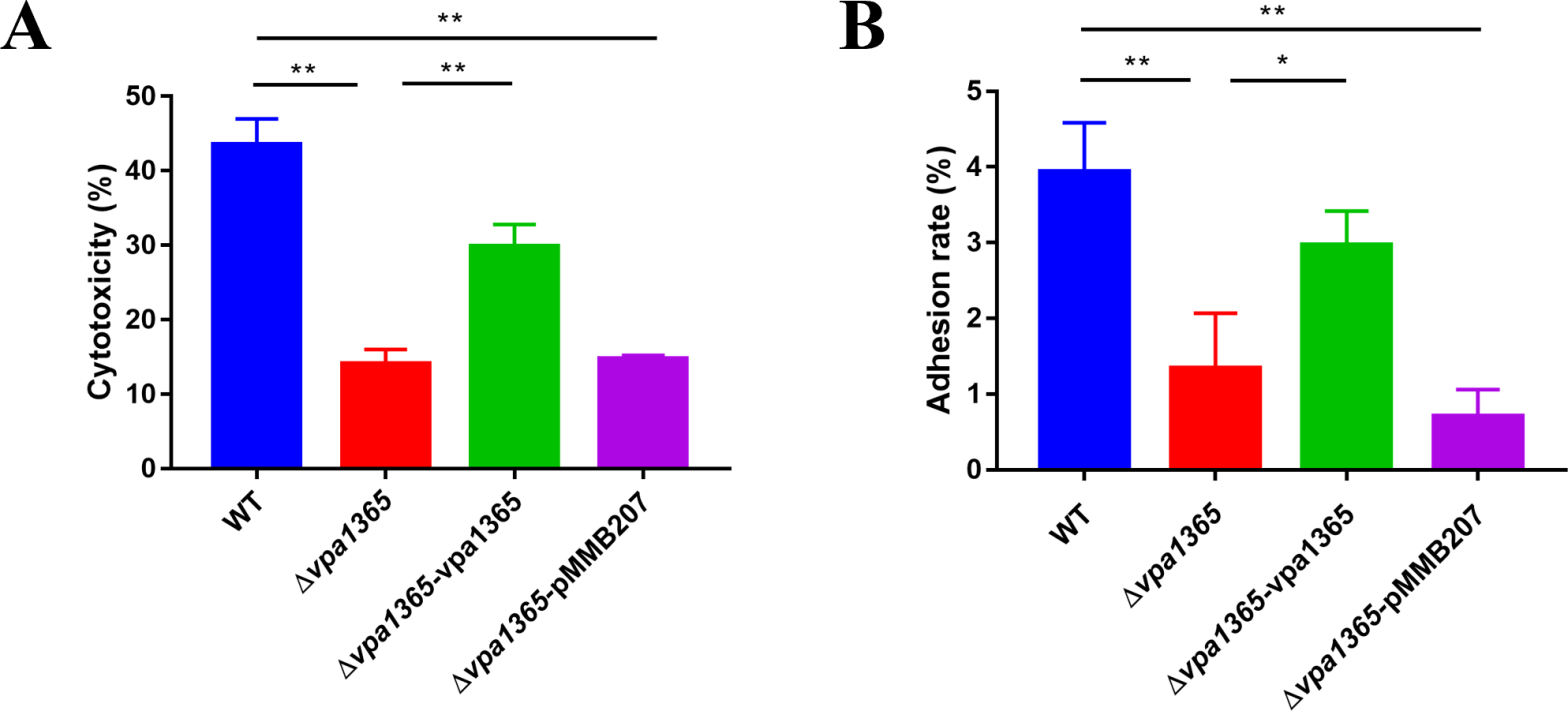
The effects of *vpa1365* on cytotoxicity and cell adhesion ability. (**A**) Cytotoxicity and (**B**) adhesion rate of HeLa cell monolayers infected with WT, ∆*vpa1365*, ∆*vpa1365-*vpa1365, and ∆*vpa1365-*pMMB207 at 2 h, respectively. Results are presented as mean ± SD (*n* = 3). Three separate experiments were performed in biological triplicates each. *P ≤ 0.05 and **P ≤ 0.01.

The main virulence factor T3SS2 is crucial for *V. parahaemolyticus* to colonize the intestine and induce massive cell death in the host by interfering with intracellular signal pathways (18, 22, 29). Therefore, we determined the cytotoxicity and adhesion rate of WT, Δ*vpa1365*, and complement strains to HeLa cells. As shown in Fig. 4, both the cytotoxicity and adhesion rate of the Δ*vpa1365* were significantly lower than those of the WT (*P* ≤ 0.01), while complementation of this gene partially restored its cytotoxicity and adhesion rate. These results revealed that *vpa1365* plays a key role in promoting adhesion ability and cytotoxicity toward HeLa cells.

### VPA1365 promotes the hemolytic activity and biofilm formation by directly binding to the promoters of scrG, pilA, and mshA

**Fig 5:**
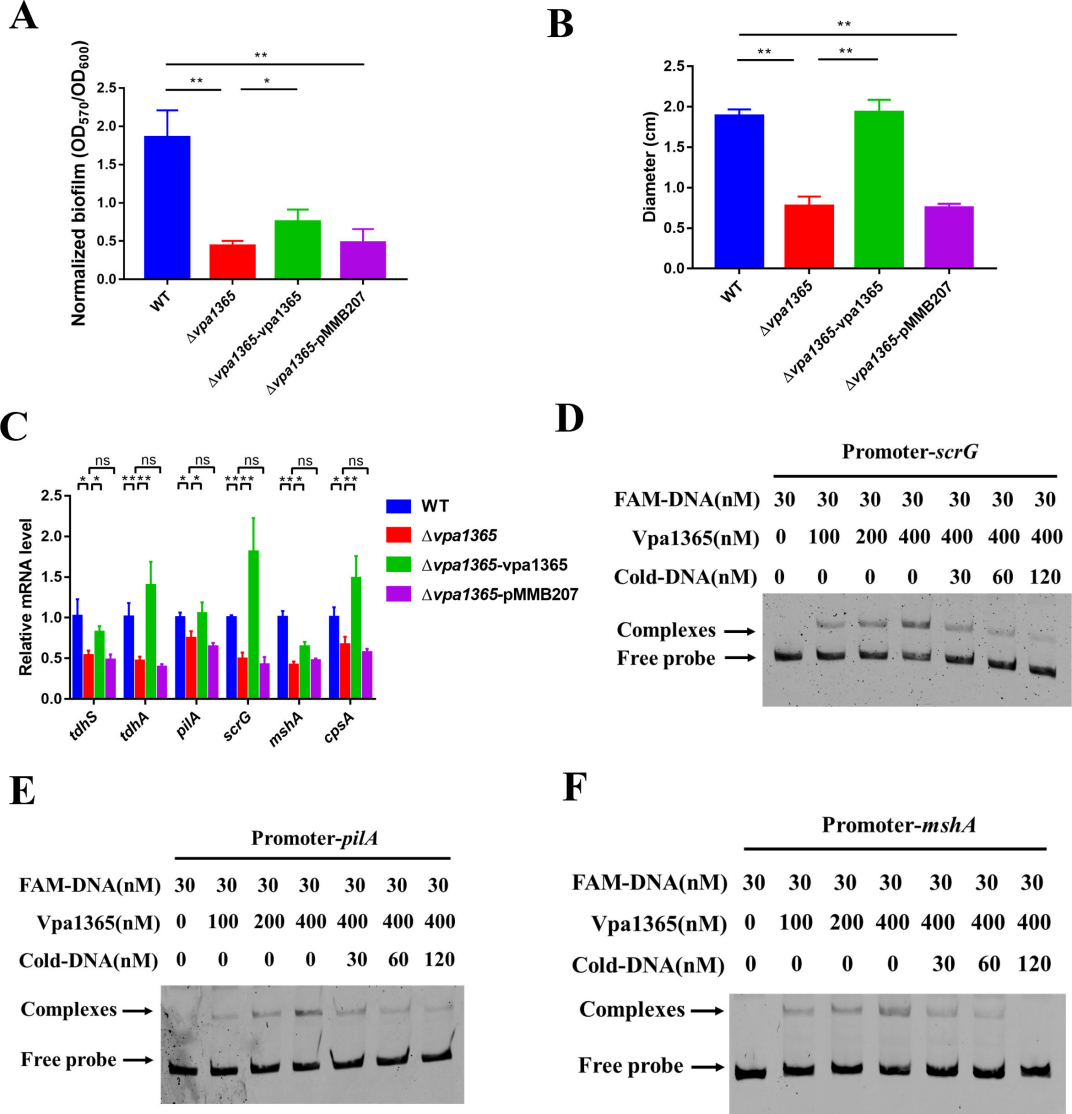
The effects of *vpa1365* on biofilm formation and hemolytic activity. (**A**) The normalized biofilm formation (total amount of biofilm/growth) of WT, Δ*vpa1365*, ∆*vpa1365-*vpa1365, and ∆*vpa1365-*pMMB207 cultured at 37°C for 31 h was stained with 0.1% crystal violet, respectively. (**B**) The hemolytic circle diameter of WT, Δ*vpa1365*, ∆*vpa1365-*vpa1365, and ∆*vpa1365-*pMMB207 growing on the Wagatsuma blood agar base. (**C**) Relative transcript levels of virulence-associated genes in WT, ∆*vpa1365*, and ∆*vpa1365-*vpa1365. (**A–C**) Results are presented as mean ± SD (*n* = 3). Three separate experiments were performed in biological triplicates each. *P ≤ 0.05 and **P ≤ 0.01. ns, no significance. (**D–F**) The analysis of binding of VPA1365 to the promoter of biofilm regulator *scrG*, type IV pilus *pilA*, and *mshA*.

Recent studies have revealed the significant role of biofilm and hemolytic activity in the pathogenicity of *V. parahaemolyticus* (50–52). Therefore, we also investigated the regulatory role of VPA1365 in these virulence factors. Our results showed that the deletion of *vpa1365* resulted in significantly lower biofilm formation than that of the Δ*vpa1365-*vpa1365 and WT (Fig. 5A; *P* ≤ 0.01). Furthermore, the diameters of hemolytic rings in Δ*vpa1365* and Δ*vpa1365-*pMMB207 were significantly smaller than those in other tested strains (Fig. 5B; *P* ≤ 0.01), indicating a marked reduction in the hemolytic activity of Δ*vpa1365*. These results revealed an important role of VPA1365 in biofilm formation and hemolytic activity.

*V. parahaemolyticus* often relies on the pili for attachment and then forms biofilm by producing polysaccharides, which is regulated by the concentration of the second messengers. The mannose-sensitive hemagglutinin type IV pili, polysaccharides, and the second messenger bis-(3′−5′)-cyclic di-GMP (c-di-GMP) have been demonstrated to play positive roles in biofilm formation (53–55). To further explore how VPA1365 affects biofilm formation and hemolytic activity, the expression levels of type IV pilin *pilA* and *mshA*, c-di-GMP metabolism enzymatic gene *scrG*, capsular polysaccharide-protein *cpsA*, thermostable direct hemolysin *tdhA*, and *tdhS* were determined in WT and Δ*vpa1365*. The results showed that VPA1365 positively regulates the expression of these virulence-associated genes (Fig. 5C). The EMSA was then used to determine whether VPA1365 directly binds to the selected promoters of *pilA*, *mshA*, *scrG*, *tdhA*, and *tdhS* genes. As expected, the VPA1365 protein directly binds to the promoters of *pilA*, *mshA*, and *scrG* in a concentration-dependent manner (Fig. 5D through 5F). The addition of cold DNA changed the shifted bands to an unshifted position. However, no binding was observed with the promoters of *tdhA* and *tdhS*, suggesting an indirect regulatory effect of VPA1365 on hemolytic activity (Fig. S3). In conclusion, our findings indicated that VPA1365 is directly bound to the promoter regions of *pilA*, *mshA*, and *scrG*, thereby regulating biofilm formation and hemolytic activity.

### Identification of VPA1365-binding motifs

**Fig 6:**
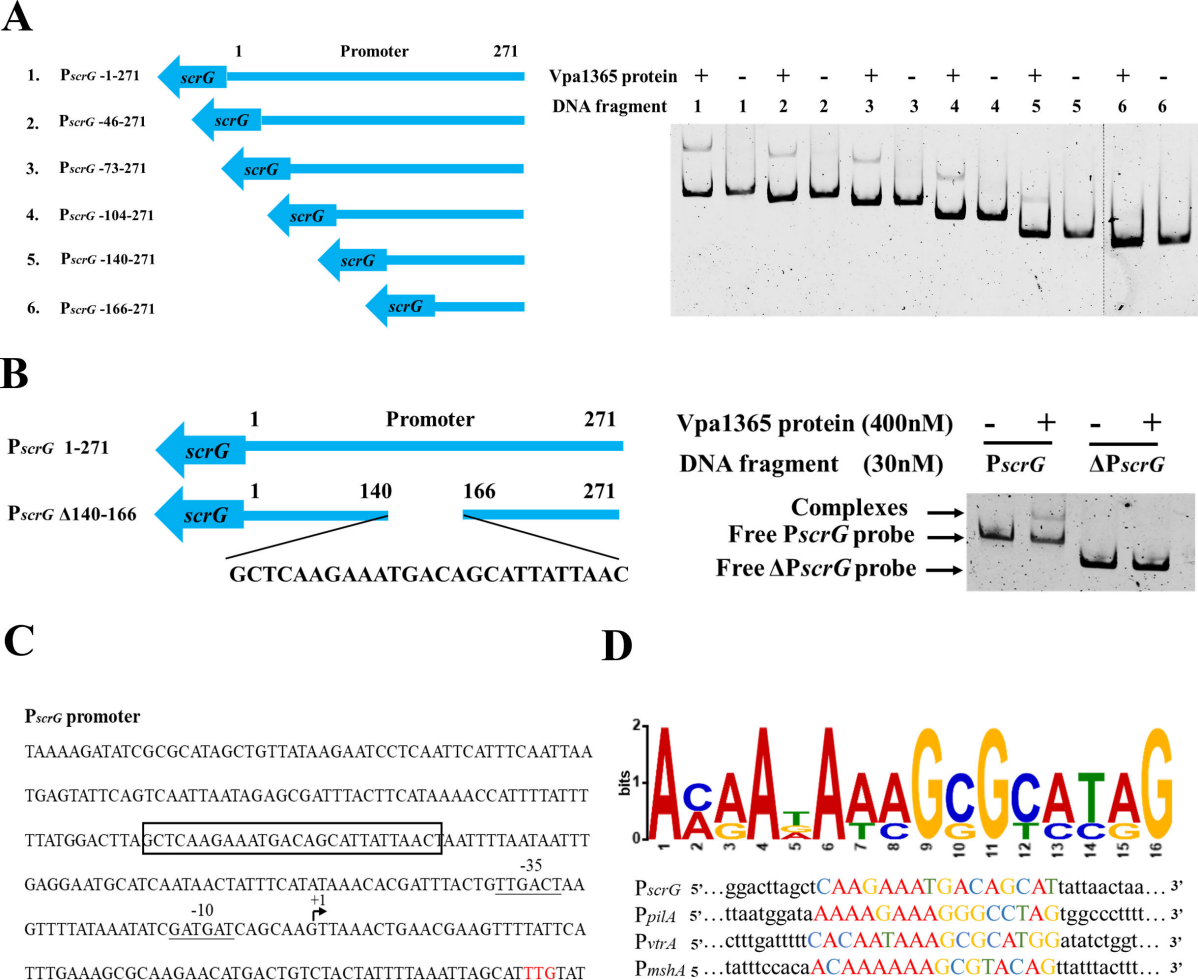
Identification of the VPA1365-binding motifs. (**A**) EMSA analysis of VPA1365 binding to the *scrG* promoter with different truncations. Six truncated fragments for the *scrG* promoter (left panel) were analyzed for their interaction with VPA1365 (right panel). (**B**) Nucleotide sequences (271 bp) of the *scrG* promoter (P*scrG*). The VPA1365-binding site is boxed in black, while −35 box and −10 box sequences are underlined. Start codon (ATG) and +1 of transcription are shown in red and with an arrow, respectively. (**C**) EMSA analysis of VPA1365 binding to the truncated ∆*scrG* promoter (∆P*scrG*) with the deletion of the VPA1365-binding site (140–166 bp). The fragment of ∆P*scrG* (left panel) was analyzed for its binding with VPA1365 (right panel). (**D**) The predicted consensus VPA1365-binding motif was generated from MEME-Suite analysis of the selected promoters.

To determine the binding sites of VPA1365, we chose the promoter of *scrG* as a representative for EMSA analysis due to its more obvious binding band. Six truncated fragments of the *scrG* promoter were tested for their interaction with VPA1365. EMSA results indicated that VPA1365 binds to DNA fragments 1, 2, 3, 4, and 5 but not to DNA fragment 6 (Fig. 6A). Subsequently, we amplified a DNA fragment with position 140–166 truncation (ΔP*scrG*) and found that VPA1365 did not bind to this fragment (Fig. 6B and C). Furthermore, using the MEME-Suite tool, we identified a putative VPA1365-binding motif within the promoters of *vtrA*, *pilA*, *mshA*, and *scrG*, designated as 5′-AAAATAAAGCGCATAG-3′. The promoter region including the transcriptional initial site of the *scrG* gene was identified by others (56). The predicted VPA1365-binding site is highly similar to the *scrG* promoter region with nucleotide positions 140–166. The transcriptional initial sites in the promoter regions of the *vtrA*, *pilA*, and *mshA* genes were identified by previous transcriptome sequencing (Fig. S4) (9, 55). Finally, we predicted and analyzed the consensus binding motif in the promoter of *scrG* and other promoters of *vtrA*, *pilA*, and *mshA* (Fig. 6D). These results further revealed that VPA1365 binds directly to the promoter and regulates the expression levels of these genes in *V. parahaemolyticus*.

### Lacking of vpa1365 attenuates virulence to *D. rerio*

**Fig 7:**
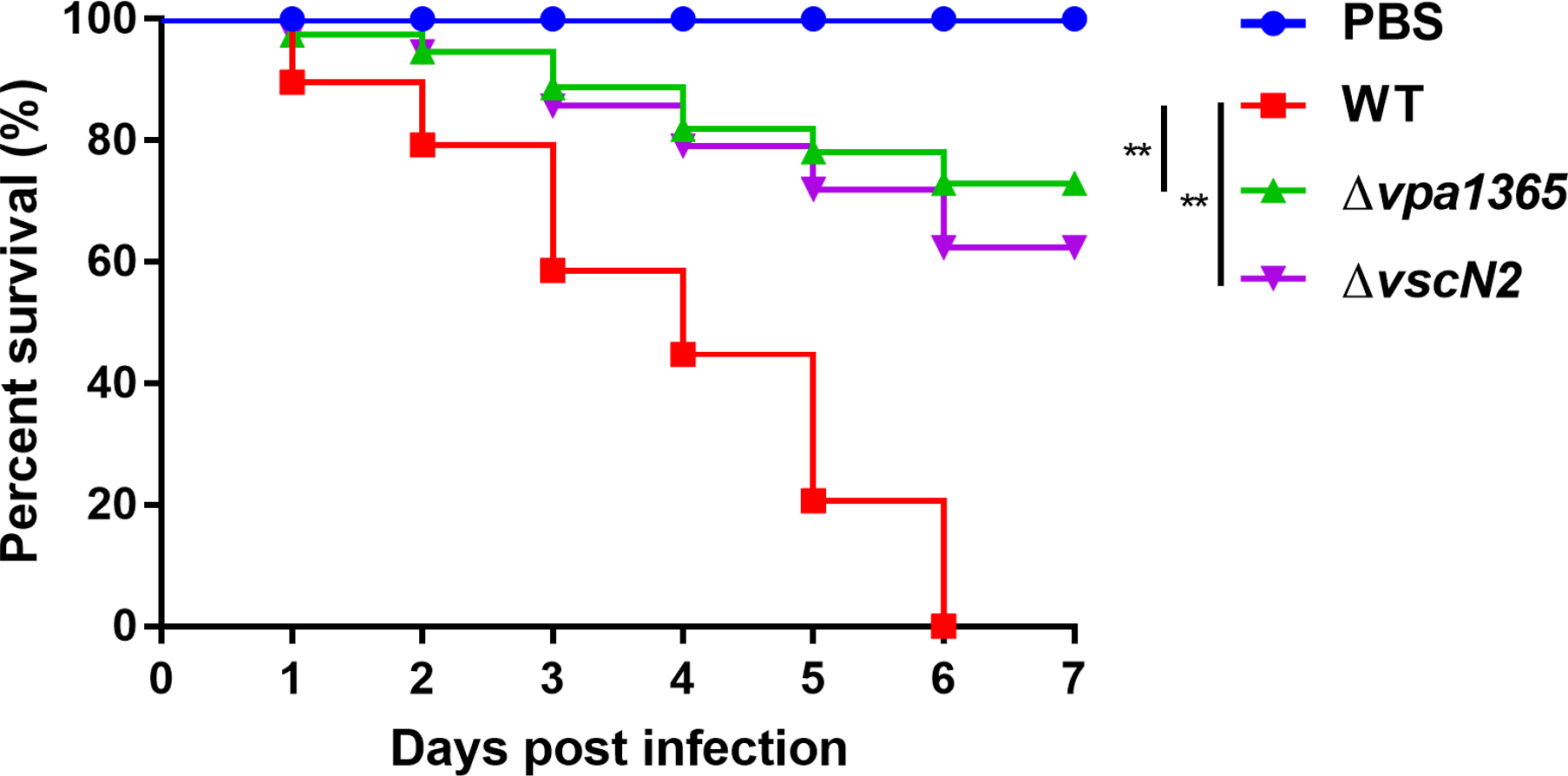
The survival rate of zebrafish challenged with WT, Δ*vpa1365*, and ∆*vscN2*, respectively. The number of dead zebrafish was recorded, and the percent survival rate in every group was calculated. The comparison of survival distribution between WT and mutant strains was performed using the log-rank (Mantel-Cox) test in GraphPad Prism version 7.0. **P ≤ 0.01.

Given the significant impairment of virulence factors in Δ*vpa1365*, we sought to determine the dependency of *V. parahaemolyticus* pathogenicity on the VPA1365. Healthy *D. rerio* (zebrafish) was infected with the WT, Δ*vpa1365*, and Δ*vscN2* strains, each at a concentration of 1 × 10^6^ CFU in the muscle. In the PBS-negative control group, no zebrafish died, whereas, in the WT-infected group, zebrafish died daily, resulting in complete mortality by day 6 (Fig. 7; *P* ≤ 0.01). The relative survival rates of zebrafish infected with Δ*vpa1365* and Δ*vscN2* were approximately 73% and 63%, respectively. These results provided additional evidence that VPA1365 is essential for the virulence of *V. parahaemolyticus*.

## DISCUSSION

Since the early discovery of the structure of the T3SS in pathogenic *Yersinia* spp. (57), two T3SSs, namely T3SS1 and T3SS2, respectively, have been identified in *V. parahaemolyticus*. The T3SS1 has been found in nearly all sequenced clinical or environmental isolates. In contrast, the occurrence of T3SS2 in pathogenic islands remains limited to pathogenic strains (11, 58), thereby suggesting an essential role for T3SS2 in the pathogenesis of *V. parahaemolyticus*. An increasing amount of evidence has indicated that the virulence genes of *V. parahaemolyticus* are stringently controlled by numerous regulators (6, 59). Previous studies have demonstrated that VtrA/B/C activates the expression of the T3SS2 gene cluster in the presence of bile salts (25). In this study, we identified and characterized a protein belonging to the TPR family, namely VPA1365, which positively regulates the function of the T3SS2. This discovery broadens the regulatory network of virulence genes and deepens our understanding of the pathogenic molecular mechanisms of *V. parahaemolyticus*.

When pathogens invade the host intestine, bile acids bind to the hydrophobic interior of the β barrel within the periplasmic spatial domains of VtrA and VtrC, thereby promoting the expression of the downstream gene *vtrB* (60). Subsequently, the activated VtrB induces the expression of T3SS2-related genes. Notably, both VtrA and VtrB belong to the OmpR family, which encompasses a DNA-binding domain featuring a winged-helix-turn-helix structure (24, 61). The transcriptional factor ToxR has also been discovered to be indispensable for the induction of *vtrB* and T3SS2 transcription (62, 63). These findings imply that the expression of T3SS2 was tightly regulated by many regulators. Moreover, Troy et al. screened 230 genes that play a vital role in the colonization of *V. parahaemolyticus* in the small intestine using Tn-seq (27). Intriguingly, among them are multiple potential transcriptional regulators, some of which have been affirmed to directly regulate the expression of flagellar-related genes and contribute to the motility of *V. parahaemolyticus* (9, 64). Therefore, we were eager to know whether these regulators have an impact on T3SS2, which has been identified as essential for the colonization in the small intestine of *V. parahaemolyticus* (27). Among them, we discovered that the protein VPA1365, located within the T3SS2 gene cluster, possesses three TPR domains and is predicted to have the potential to interact with other nucleotides. Our study subsequently confirmed that the deletion of *vpa1365* significantly reduced the virulence and colonization ability of *V. parahaemolyticus* in infant rabbits (*P* ≤ 0.01). Thus, we further hypothesized that VPA1365 might affect the colonization of *V. parahaemolyticus* in the rabbit intestine by regulating the function of T3SS2.

The TPR domain contains two antiparallel α-helix subunits that display a right-handed supercoil structure, enhancing the ability of the channel to effectively interact with the target protein (65, 66). More recently, it was found that TPR domains are present in numerous DNA- and RNA-binding proteins (45–48). In a previous study, the T3SS2 protein PthA containing a TPR-like domain in *Xanthomonas axonopodis* pv. *citri* was shown to undergo conformational changes upon DNA interaction, similar to pentatricopeptide repeat domain, a nucleic acid-binding motif, further revealing its nucleic acid binding ability (65). Until now, the TPR family is widely associated with diverse biological processes, including metabolic regulation, gene expression, and protein export (66). The biological properties of many TPR family genes have been extensively studied in plants like *Solanum lycopersicum* and *Arabidopsis* (67, 68); unfortunately, their roles in microorganisms remain relatively limited. In avian pathogenic *E. coli*, the T3SS chaperone protein YgeG possessing TPR domains has an impact on virulence and pathogenicity (69). In *Borrelia burgdorferi*, a putative TPR domain is of great significance for the function of the hypothetical protein BB0238, and mutation of key residues in the α-helix significantly reduces tissue infection rates in the C3H/HeN mice infection model (70). Furthermore, two diguanylate cyclase genes, *scrJ* (VPA1115) and *scrL* (VPA1069), were identified to contain conserved TPR domains and coupled GGDEF proteins, contributing to the surface colonization of *V. parahaemolyticus* (71). Current studies have demonstrated a close connection between the TPR domain and virulence in pathogens, warranting further exploration of its molecular pathogenic mechanism. Our study found that VPA1365, containing three conserved TPR domains, directly binds to the promoter regions of the T3SS2 master regulatory factor *vtrA* (Fig. 3). Both qRT-PCR and immunoblotting results further confirmed that the absence of *vpa1365* decreased the expression levels of T3SS2-related genes and the secretion of VopD2 (Fig. 3). Therefore, we proposed that VPA1365 directly binds to the *vtrA* promoter and then regulates the expression of *vtrB* and T3SS2-related genes.

Interestingly, overexpression of the T3SS2 positive regulator VtrA or VtrB in Δ*vpa1365* not only upregulates the expression level of T3SS2-related genes but also increases the secretion of VopD2 into the supernatant under bile salts conditions. These results suggested that VPA1365 may regulate the expression of T3SS2 through VtrA/VtrB, warranting further exploration of the relationship between VPA1365 and VtrA/VtrB. Furthermore, environmental factors also influence the expression of T3SS2, and we found that the absence of VPA1365 completely abolishes the expression and secretion of VopD2 under low NaCl conditions (Fig. 3). This finding aligns with our previous discovery that low NaCl affects the T3SS2 function in *V. parahaemolyticus* (30), indicating that low NaCl might serve as a signal influencing VPA1365-mediated regulation of T3SS2. However, under low NaCl conditions, overexpression of either VtrA or VtrB in the Δ*vpa1365* did not restore VopD2 secretion to levels comparable to those in WT and Δ*vpa1365-*vpa1365, further highlighting the importance of VPA1365 in positively regulating the function of T3SS2 in response to low NaCl. Additionally, the high conservation of VPA1365 in the different *Vibrio* species suggests that this regulatory mechanism may be widespread among other *Vibrio* species.

It is generally considered that *V. parahaemolyticus* T3SS1 is mainly associated with cytotoxicity, while T3SS2 is primarily connected to enterotoxicity (16). However, previous studies have revealed that VtrA/VtrB is also of vital importance for T3SS2-dependent cytotoxicity (72, 73). The comparative genome analysis indicated that the Vp-PAI encompasses not only the T3SS2 gene cluster but also two *tdh* genes (*tdhA* and *tdhS*) (15, 74). Deletion of *vtrA* or *vtrB* decreased the production of TDH, the major virulent determinant influencing cytotoxicity and enterotoxicity, in *V. parahaemolyticus* (20, 75). Additionally, motility, biofilm formation, and adhesion ability play important roles in the infection process of *V. parahaemolyticus* (76). Therefore, we sought to determine whether VPA1365 has an impact on these crucial virulence factors. In comparison to the WT, deletion of *vpa1365* significantly reduced cytotoxicity, adhesion rate, biofilm formation, and hemolytic activity (Fig. 4, Fig. 5A and B), while it had no effect in affecting the swimming and swarming motility (Fig. S5). The biofilm formation in Δ*vpa1365-*vpa1365 did not fully restore to the level of WT, possibly because overexpression of VPA1365 might also regulate the other regulators of biofilm formation. The EMSA results revealed an interesting finding: the VPA1365 directly binds to the promoter regions of type IV pilin *mshA*, *pilA*, and biofilm regulatory protein *scrG*. However, it is unable to directly bind to the promoter regions of the thermostable direct hemolysin *tdhA* and *tdhS*. These results suggested that VPA1365 might act as a global regulatory factor to influence the expression level of various virulence genes. Moreover, it may indirectly regulate hemolysin activity by promoting the expression level of T3SS2 regulator *vtrA* or other transcriptional factors. Based on the truncated EMSA analysis and the MEME-Suite tool, we predicted a putative VPA1365-binding motif within the promoters of *vtrA*, *pilA*, *mshA*, and *scrG* designated as 5′-AAAATAAAGCGCATAG-3′. This motif bears some resemblance to the previously reported DNA sequence bound with a TPR-like protein PthA, which is a TA-rich region (65). Interestingly, the *nuc2*+ protein containing the TPR domain in *Schizosaccharomyces pombe* is also capable of binding AT-rich DNA *in vitro* (46). These findings suggested that the fragments containing AT-rich regions are likely to be the fragments of proteins bound with the TPR domain. Finally, considering the relatively weak interactions of VPA1365 with these promoters and the involvement of the TPR domain in protein-protein interactions, VPA1365 may exert its effect on transcription in an alternative manner, such as through interactions with other nucleoid-binding proteins like H-NS. Regardless, the positive regulatory effect of VPA1365 on the function of T3SS2 and virulence-related genes is unquestionable. However, whether VPA1365 interacts with other DNA-binding proteins to co-regulate the expression of T3SS2 remains to be explored in the future.

Zebrafish commonly serve as an ideal model to investigate the virulence mechanisms employed by both Gram-negative and Gram-positive pathogens that affect fish and humans (77). Currently, zebrafish have been employed as an animal model to assess the virulence of *V. anguillarum*, *V. cholerae*, and *V. parahaemolyticus* (44, 78, 79). Therefore, we used the zebrafish as an infection model to evaluate the influence of VPA1365 on the virulence of zoonotic *V. parahaemolyticus* in fish and humans. The survival rates of zebrafish infected with Δ*vpa1365* and Δ*vscN2* (Δ*vscN2* serving as a T3SS2 functional reference) were approximately 73% and 63%, respectively, whereas all zebrafish died by day 6 in the WT-infected group. Notably, the survival rate of Δ*vpa1365* was significantly higher than that of Δ*vscN2*, suggesting that the impact of the regulatory factor VPA1365 on virulence includes but is not limited to the T3SS2. Nevertheless, the deletion of *vpa1365* significantly reduced the lethality and colonization ability of *V. parahaemolyticus* in infant rabbits and zebrafish.

### Conclusion

**Fig 8:**
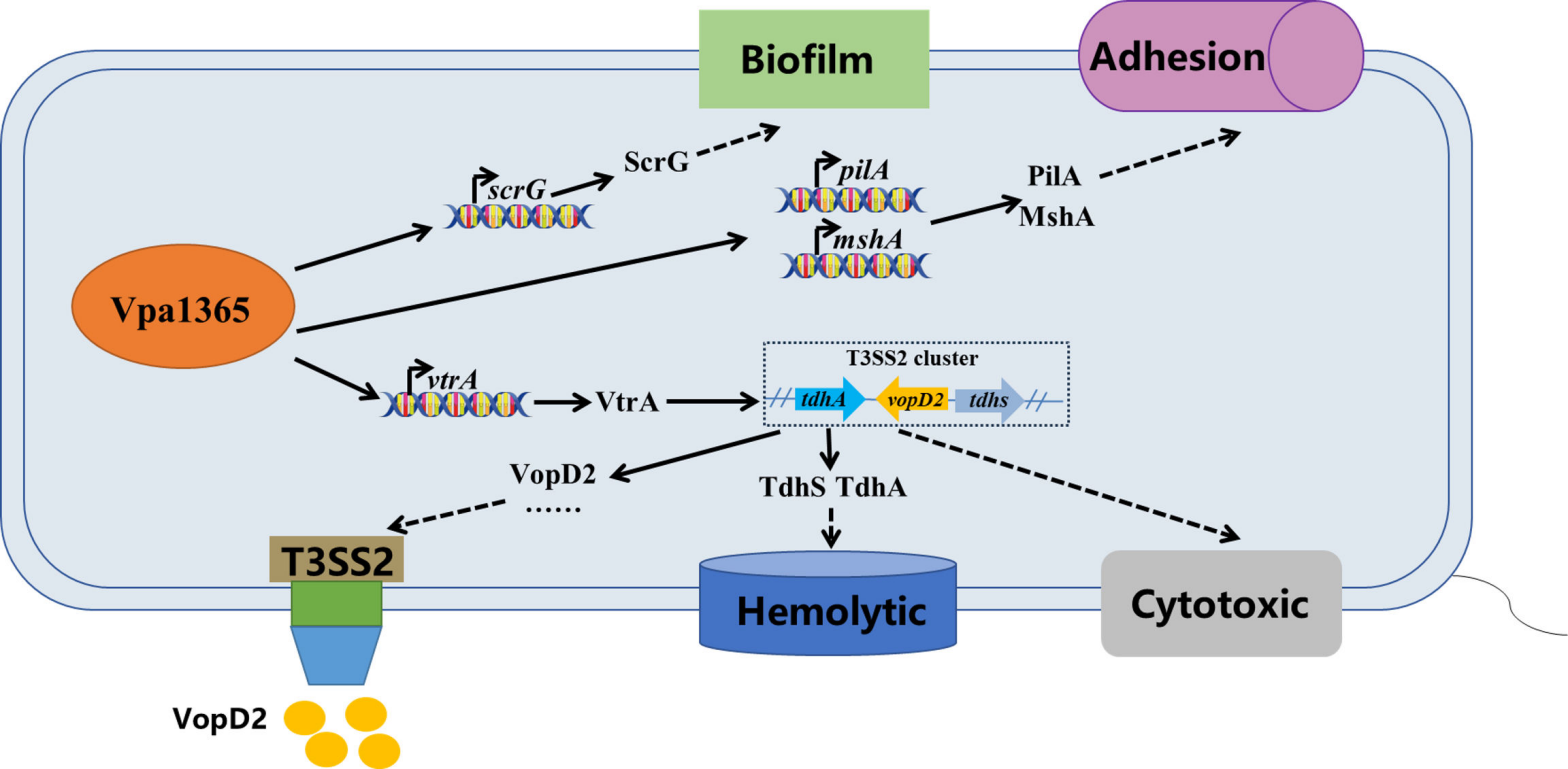
The putative model of VPA1365 regulation in *V. parahaemolyticus*. VPA1365 indirectly contributes to the function of T3SS2 by binding to the promoter of *vtrA* and additionally positively regulates hemolytic activity, biofilm formation, cytotoxicity, and adhesion ability. The black solid arrows indicate direct binding or regulation, and the black dashed arrows indicate indirect or unclear regulation.

In the present study, we identified a protein belonging to the TPR family, namely VPA1365, which is located within the T3SS2 gene cluster. This particular protein directly binds to the promoter regions to regulate the expression of T3SS2, thereby exerting a significant influence on the intestinal colonization of *V. parahaemolyticus*. Additionally, VPA1365 functions as a global regulator, modulating the hemolytic activity, biofilm formation, adhesion ability, and cytotoxicity of *V. parahaemolyticus*. Its regulation of biofilm formation and adhesion likely occurs by directly binding to the promoter regions of *pilA*, *mshA*, and *scrG* (Fig. 8). Overall, this study established the regulatory networks of VPA1365 and its contribution to diverse virulence factors of *V. parahaemolyticus*.

## SUPPLEMENTAL MATERIAL

Supplemental figures aem.02201-24-s0001.docx Figures S1 to S5.

Table S1 aem.02201-24-s0002.docx Primers used for cloning, EMSA, and real-time quantitative RT-PCR.

ASM does not own the copyrights to Supplemental Material that may be linked to, or accessed through, an article. The authors have granted ASM a non-exclusive, world-wide license to publish the Supplemental Material files. Please contact the corresponding author directly for reuse.
