# Supplementary material for: Role of the TPR family protein VPA1365 in regulating type III secretion system 2 and virulence in Vibrio parahaemolyticus
Source: Appl Environ Microbiol. 2025 Mar 25;91(4):e02201-24. doi: 10.1128/aem.02201-24 (PMC12016518; doi:10.1128/aem.02201-24)
Supplement: Supplemental figures — Figures S1 to S5. [file aem.02201-24-s0001.docx]

**Figure.S1.** The growth of WT, Δ*vpa1365*, Δ*vpa1365-*vpa1365, Δ*vpa1365-*pMMB207, Δ*vpa1365-*vtrA and Δ*vpa1365-*vtrB shaken in LB medium at 37 °C for 10 hours. Results are presented as mean ± SD (n = 3). The experiments were performed three independent times.

**Figure.S2.** The analysis of binding of VPA1365 to the promoter of additional virulence-associated genes. EMSA was performed to determine the binding of VPA1365 to the promoter of the *gyrB* (A), *vtrB* (B), *vpa1364* (C) and *vpa1380* (D). The promoter of *gyrB* was used as a negative control.

**Figure.S3.** The analysis of binding of VPA1365 to the promoter of virulence-associated hemolysin genes. EMSA was performed to determine the binding of VPA1365 to the promoter of thermostable direct hemolysin *tdhA* (A) and *tdhS* (B).

**Figure.S4.** Nucleotide sequences of *vtrA* (A), *pilA* (B) and *mshA* (C) promoters. The predicted VPA1365-binding site is enclosed in a black box, -35 box, and -10 box sequences are underlined. Start codon (ATG) and +1 of transcription is shown in red and with an arrow, respectively.

**Figure.S5.** The effects of *vpa1365* on motility. The swarming and swimming rings diameter of WT, Δ*vpa1365*, Δ*vpa1365-*vpa1365 and Δ*vpa1365-*pMMB207 of continuous culturing on the 1.5% BHI (A) and 0.3% LB (B) agar plates at room temperature for 18 h, respectively. Results are presented as mean ± SD (n = 3). The experiments were performed three independent times.


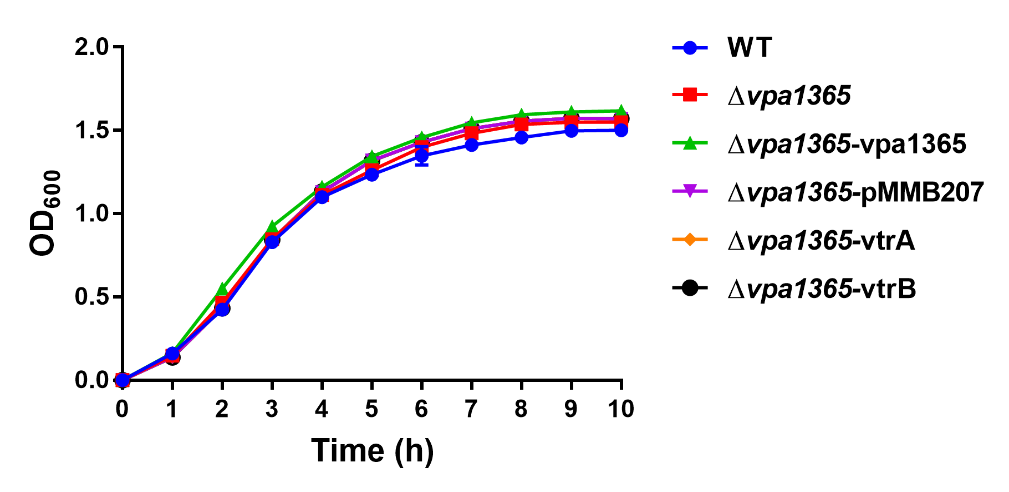


**Fig.S1.**

A. B.


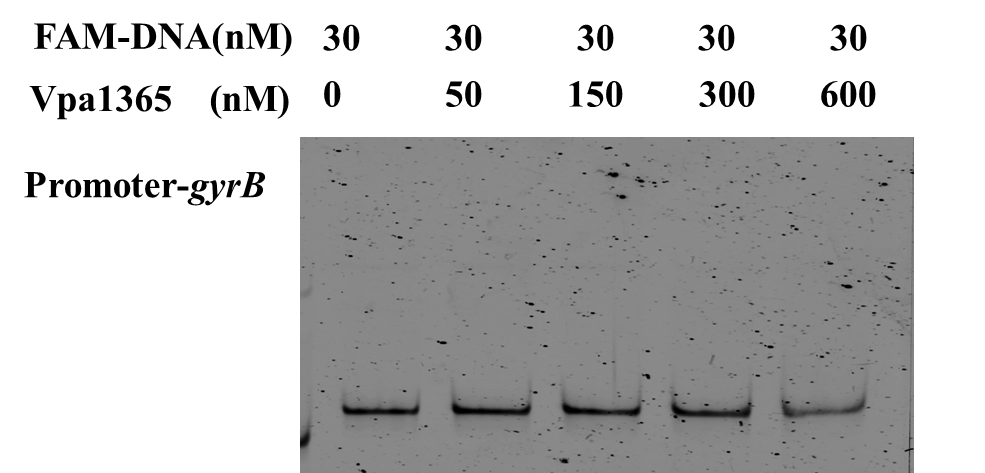

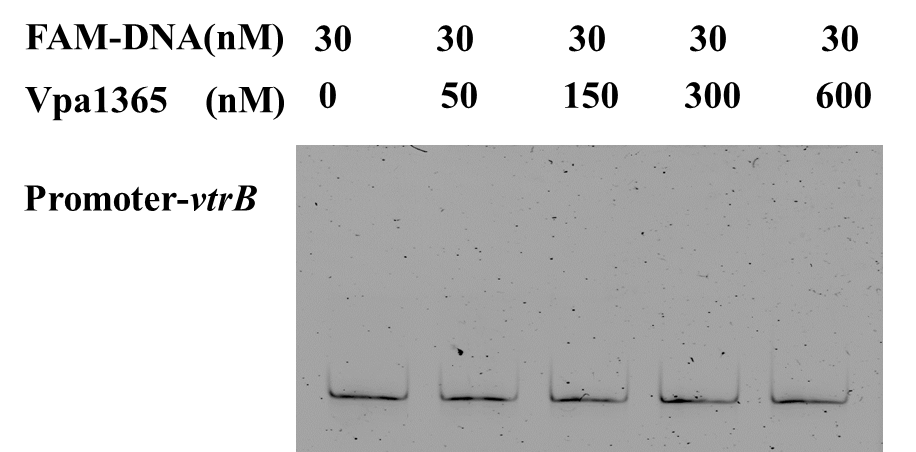


C. D.


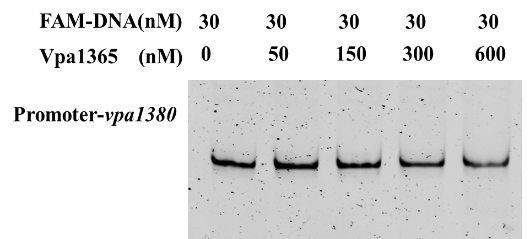

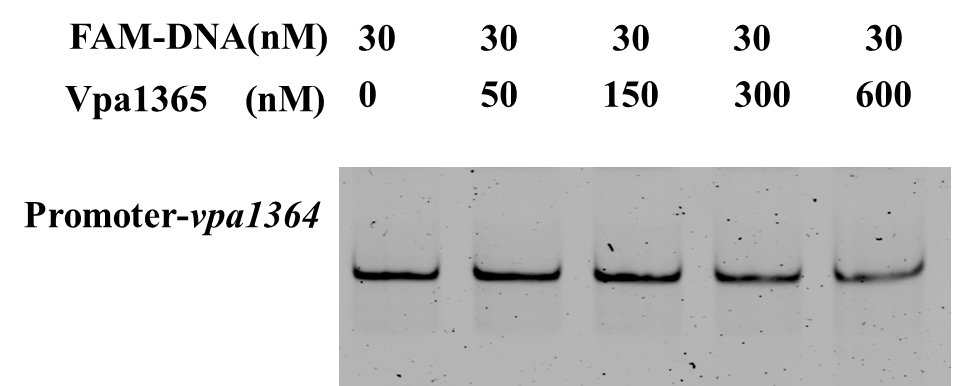


**Fig.S2.**

1. B.


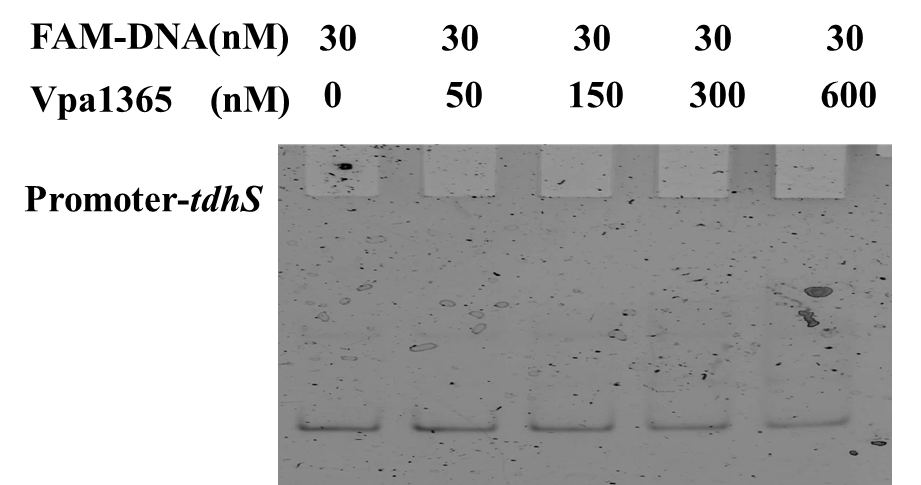

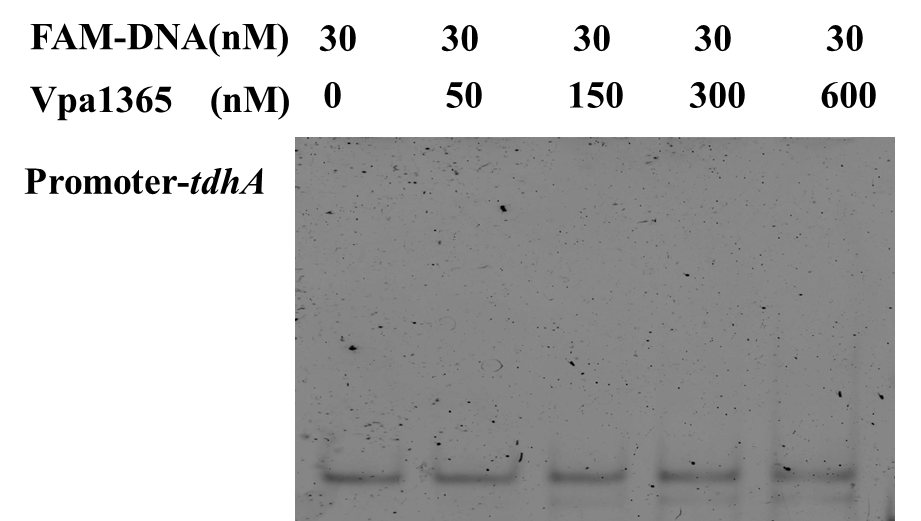


**Fig.S3.**

**
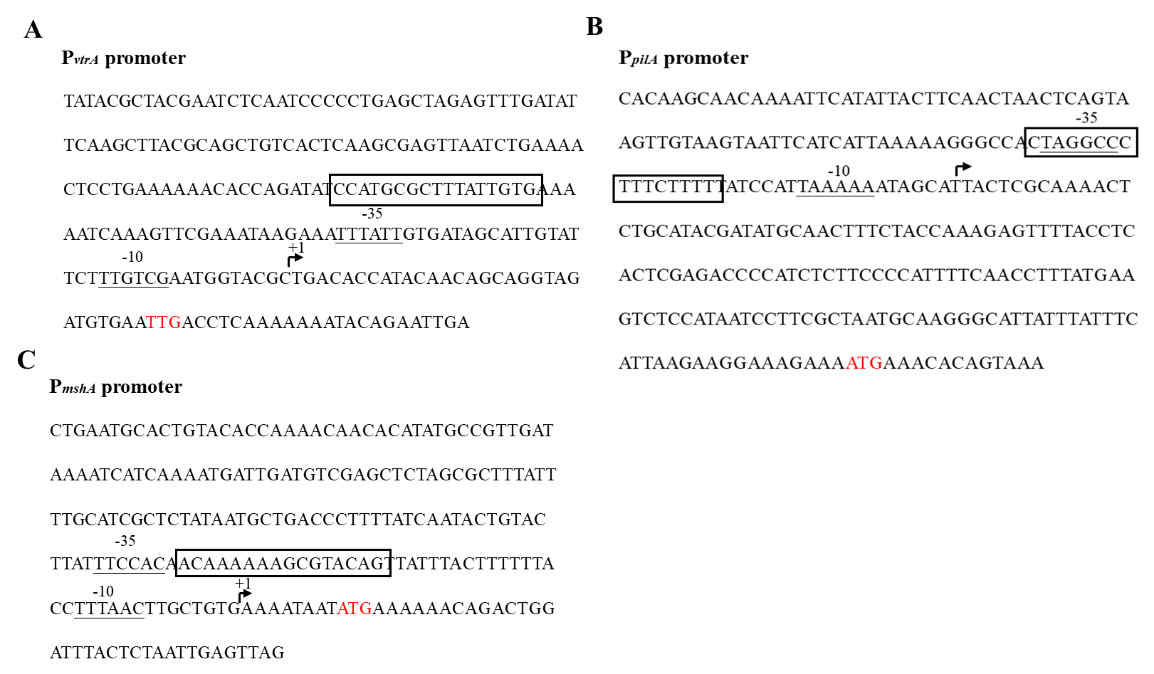
**

**Fig.S4.**


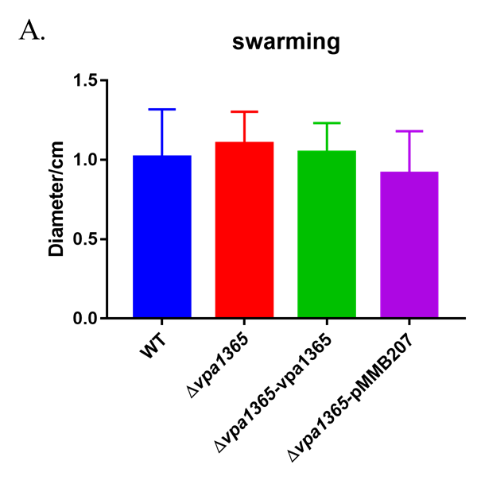

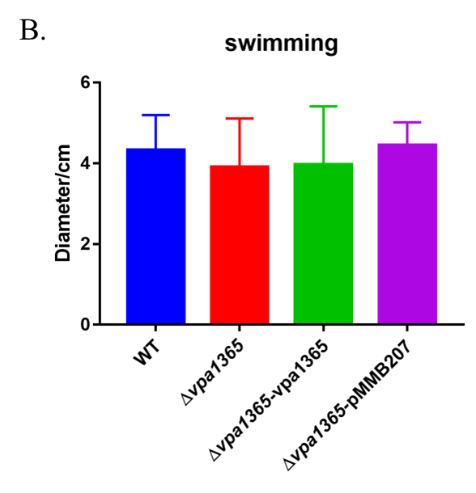


**Fig.S5.**
