# Supplementary material for: Role of the TPR family protein VPA1365 in regulating type III secretion system 2 and virulence in Vibrio parahaemolyticus
Source: Appl Environ Microbiol. 2025 Mar 25;91(4):e02201-24. doi: 10.1128/aem.02201-24 (PMC12016518; doi:10.1128/aem.02201-24)
Supplement: Table S1 — Primers used for cloning, EMSA, and real-time quantitative RT-PCR. [file aem.02201-24-s0002.docx]

**Table S1.** Primers used for cloning, EMSA, and real-time quantitative RT-PCR.

| Primer name | Primer sequence (5’ to 3’) | Target | |
| --- | --- | --- | --- |
| *vscN2*-UF | GAGCGGATAACAATTTGTGGAATCCCGGGAGGTATTGCTCGTAGTGTCT | *vscN2* | |
| *vscN2*-UR | TCTTCATACTCCTTGATACGACTCCTTCAC | *vscN2* | |
| *vscN2*-DF | TCGTATCAAGGAGTATGAAGAAACCCTCC | *vscN2* | |
| *vscN2*-DR | AGCGGAGTGTATATCAAGCTTATCGATACCCCTGATCAAGTACAAAGCT | *vscN2* | |
| *vscN2-*out*-*F | GACCAACCTAGTGGCATTG | Test*-vscN2* | |
| *vscN2-*out*-*R | TCACCGTCTACCGTATCTC | Test-*vscN2* | |
| *vscN2-*in-F | ATCGCCGTTGAGCCTATTCC | Test*-vscN2* | |
| *vscN2-*in-R | CTGGACCGATTTCCCCTTCA | Test-*vscN2* | |
| *Vpa1365*-UF | GATAACAATTTGTGGAATCCCGGGACAAATACACTGCGTGCAGACCAGAC | *vpa1365* | |
| *vpa1365*-UR | TCAAGTGAGCGAGGCTGCAGCAGTCTTCATTATC | *vpa1365* | |
| *vpa1365*-DF | AGTGTATATCAAGCTTATCGATACCTTAGTTACTTCGTTCAGGTGGTG | *vpa1365* | |
| *vpa1365*-DR | CTGCAGCCTCGCTCACTTGATTACTCTCTCAAAG | *vpa1365* | |
| *vpa1365*-out-F | TTGGCTTTACCTAACGCCTCATC | Test*-vpa1365* | |
| *vpa1365*-out-R | GCAGTTTGAAGTGCTCACAGGGT | Test*-vpa1365* | |
| *vpa1365*-in-F | ACTGCTCGATTTAGGTGATGCT | Test*-vpa1365* | |
| *vpa1365*-in-R | ATTGAGCATGTCCAACGCCT | Test*-vpa1365* | |
| *vpa1365*-F | GCGTCGACTCAGTGATGATGATGATGTATCAAATATGGATTTAACTGTTTGCG | 207*-vpa1365* | |
| *vpa1365-*R | CCGCGAGCTCTAAGGAGGTAGGATAATAGTGAGCGAATTTGAAATAGAAG | 207*-vpa1365* | |
| pMMB207-F | GAGCGGATAACAATTTCACACAGG | Test-pMMB207 | |
| pMMB207-R | GATTTAATCTGTATCAGG | Test-pMMB207 | |
| EMSA-*vtrA*-F | TGCCTGCAGGTCGACGATCACAATCTTAGATCTTCTTTTGCAG | Promotor *vtrA* | |
| EMSA-*vtrA*-R | TTCACATCTACCTGCTGTTGTATGG |  | |
| EMSA-*vtrB*-F | TGCCTGCAGGTCGACGATCGCTGAGCCCTTTTCACAGTTTTTC | Promotor *vtrB* | |
| EMSA-*vtrB*-R | TTCACATCTACCTGCTGTTGTATGG |  | |
| EMSA-*vpa1342*-F | TGCCTGCAGGTCGACGATACAACAAAAAGCACAAGCT | Promotor *vpa1342* | |
| EMSA-*vpa1342*-R | AGACGAGATGTTTTATCATCGTATT |  | |
| EMSA-*tdhA*-F | TGCCTGCAGGTCGACGATATGGCCATGTTACCGCTTGA | Promotor *tdhA* | |
| EMSA-*tdhA*-R | TGCCAGAATGGCAGGTTTCA |  | |
| EMSA-*tdhS*-F | TGCCTGCAGGTCGACGATAGCGGTACGGCTATCATAAA | Promotor *tdhS* | |
| EMSA-*tdhS*-R | GTGAAAAATGCTTTCTCAAACCT |  | |
| EMSA-*pilA*-F | TGCCTGCAGGTCGACGATCACAAGCAACAAAATTCATATTA | Promotor *pilA* | |
| EMSA-*pilA*-R | CTTTCCTTCTTAATGAAATAAATAATGC |  | |
| EMSA-*scrG*-F | TGCCTGCAGGTCGACGATAGTCATGTTCTTGCGCTTTCA | Promotor *scrG* | |
| EMSA-*scrG*-R | GCGCATAGCTGTTATAAGAATCC |  | |
| EMSA-*mshA*-F | TGCCTGCAGGTCGACGATATTATTTTCACAGCAAGTTAAAG | Promotor *mshA* | |
| EMSA-*mshA*-R | CGCTCTATAATGCTGACCCT |  | |
| EMSA-*gyrB*-F | TGCCTGCAGGTCGACGATTGCCTGCAGGTCGACGATGCGCGCG | Promotor *gyrB* | |
| EMSA-*gyrB*-R | TGCCAGCGCACCGCTGACCGCAG |  | |
| qPCR-*vpa1380*-F | TAGTCACGGCTGCCAAAAGT | *vpa1380* | |
| qPCR-*vpa1380*-R | CCTGATGAACCATGGCCTGT |  | |
| qPCR-*vopD2*-F | CTGCAAGGAGTGCAAGCAAC | *vopD2* | |
| qPCR-*vopD2*-R | AGCGCAACCAGTTATTCCAGA |  | |
| qPCR-*vpa1342*-F | CCTGTTGGTATTGGTGCTGC | *vpa1342* | |
| qPCR-*vpa1342*-R | CAGAGCCGCCAATAGGTTGA |  | |
| qPCR-*vtrB*-F | CTTAGGTGGGCGTGAAGCAG | *vtrB* | |
| qPCR-*vtrB*-R | TACCCCACACTTTGTCGTTG |  | |
| qPCR-*vtrA*-F | TGCACAAAAAGCCTATGCTCG | *vtrA* | |
| qPCR-*vtrA*-R | CGTGGCTAACCCGTTAGGAT |  | |
| qPCR-*vpa1364*-F | GTGCCAAGGAAGGGCTACTT | *vpa1364* | |
| qPCR-*vpa1364*-R | GTTGAACAAGCCAATCCGCA |  | |
| qPCR-*tdhA*-F | GGCTGCATTCAAAACATTTGCC | *tdhA* | |
| qPCR-*tdhA*-R | TACATTGACCGGTGCATTGGTATTA |  | |
| qPCR-*tdhS*-F | GGCTGCATTCAAAACATCTGCT | *tdhS* | |
| qPCR-*tdhS*-R | CATTGACCGGAGCTTGGGTATTA |  | |
| qPCR-*pilA*-F | TACACCGCCACCCATAACG | *pilA* | |
| qPCR-*pilA*-R | AGCCATTCTCGCCAGGTATG |  | |
| qPCR-*scrG*-F | AAGCCGTGGTGGAAGAAGG | *scrG* | |
| qPCR-*scrG*-R | GCGTGTTGAGTGCGTTGG |  | |
| qPCR-*cpsA*-F | GAGAGCGGCAACCTATATCG | *cpsA* | |
| qPCR-*cpsA*-R | CGCCACGCCAACAGTAATG |  | |
| qPCR-*mshA*-F | GCGATTGATGGTGCTTCTG | *mshA* | |
| qPCR-*mshA*-R | GCCCAATCTTCATCCAAACC |  | |
| qPCR-*gyrB*-F | TTACCGTCATGGTGAGCCTG | *gyrB* | |
| qPCR-*gyrB*-R | CACGCAGACGTTTTGCTAGG |  | |
|  |  | |  |
